# Exogenous alpha 1-antitrypsin down-regulates SERPINA1 expression

**DOI:** 10.1371/journal.pone.0177279

**Published:** 2017-05-09

**Authors:** Ahmad Karadagi, Helene Johansson, Helen Zemack, Sandeep Salipalli, Lisa-Mari Mörk, Kristina Kannisto, Carl Jorns, Roberto Gramignoli, Stephen Strom, Knut Stokkeland, Bo-Göran Ericzon, Danny Jonigk, Sabina Janciauskiene, Greg Nowak, Ewa C. S. Ellis

**Affiliations:** 1 Division of Transplantation Surgery, Department of Clinical Science, Intervention and Technology (CLINTEC), Karolinska Institute, Karolinska University Hospital Huddinge, Stockholm, Sweden; 2 Department of Respiratory Medicine, Research in Endstage and Obstructive Lung Disease Hannover (BREATH), Member of the German Center for Lung Research (DZL), Hannover Medical School, Hannover, Germany; 3 Division of Pathology, Department of Laboratory Medicine, Karolinska Institute, Karolinska University Hospital Huddinge, Stockholm, Sweden; 4 Department of Medicine, Visby Hospital, Visby, Sweden; 5 Department of Medicine, Gastroenterology and Hepatology Unit, Karolinska Institute, Stockholm, Sweden; 6 Institute of Pathology, Hannover Medical School, Hannover, Germany; Medizinische Fakultat der RWTH Aachen, GERMANY

## Abstract

The main goal of the therapy with purified human plasma alpha1-antitrypsin (A1AT) is to increase A1AT levels and to prevent lungs from elastolytic activity in patients with PiZZ (Glu^342^Lys) A1AT deficiency-related emphysema. Potential hepatic gains of this therapy are unknown. Herein, we investigated the effect of A1AT therapy on SERPINA1 (gene encoding A1AT) expression. The expression of SERPINA1 was determined in A1AT or A1AT plus Oncostatin M (OSM) treated primary human hepatocytes isolated from liver tissues from A1AT deficient patients and control liver tissues. In addition, SERPINA1 mRNA was assessed in lung tissues from PiZZ emphysema patients with and without A1AT therapy, and in adherent human peripheral blood mononuclear cells (PBMC) isolated from healthy PiMM donors. In a dose-dependent manner purified A1AT lowered SERPINA1 expression in hepatocytes. This latter effect was more prominent in hepatocytes stimulated with OSM. Although it did not reach statistical significance (P = 0.0539)—analysis of lung tissues showed lower SERPINA1 expression in PiZZ emphysema patients receiving augmentation therapy relative to those without therapy. Finally, exogenously added purified A1AT (1mg/ml) reduced SERPINA1 expression in naïve as well as in lipopolysaccharide (LPS)-stimulated human adherent PBMCs. Exogenous A1AT protein reduces its own endogenous expression. Hence, augmentation with native M-A1AT protein and a parallel reduction in expression of dysfunctional mutant Z-A1AT may be beneficial for PiZZ liver, and this motivates further studies.

## Introduction

Alpha 1-antitrypsin (A1AT) is an acute phase protein, highly expressed during inflammatory processes and one of the major proteinase inhibitors predominantly synthesized by hepatocytes [1].

The A1AT protein is encoded by the protease inhibitor (Pi) locus on chromosome 14q32.1, as a part of gene cluster called SERPIN supergene [1, 2]. The Z variant of A1AT, which differs from the normal M variant by substitution of Glu342 with Lys (6), is the most prevalent A1AT deficiency (A1ATD) variant among Caucasians, associated with risk for developing early onset lung emphysema and liver disease at any age. The incidence of ZZ A1ATD is 1 in 1 500–5 000 individuals, suggesting that approximately 100 000 subjects in western countries are affected by this mutation [3, 4].

Severe PiZZ A1ATD is associated with about 90% lower level of plasma A1AT (normal level 1–2 mg/ml) that arise not from the lack of protein synthesis but from its intracellular polymerization and accumulation [2]. Various factors can influence the rate of Z-A1AT intracellular and extracellular polymerization, and the retained Z-A1AT polymers in hepatocytes are suggested to contribute to and/or cause liver cell damage with a variable clinical presentation, from neonatal hepatitis to liver cirrhosis with increased risk of hepatocellular carcinoma in adults (10).

A1ATD is an accepted indication for both lung and liver transplantation [3–7]. However, hepatic damage is often overlooked in early preventable stages. Due to organ shortage, A1ATD patients are often not considered for liver transplantation until the manifestation of liver damage and are transplanted only when there is extensive liver disease. Hepatic involvement is mostly considered in neonates as these patients often present with cholestasis and considerable morbidity. However, the acutely ill neonate populations only accounts for a small fraction of A1ATD patients. The majority goes on unnoticed while up to 15% develop cirrhosis and hepatocellular carcinoma as reported by the Swedish autopsy studies [8, 9].

The only specific treatment for patients with A1ATD-related emphysema is intravenous infusions of plasma purified A1AT [4]. The concept behind this therapy is to raise the concentration of A1AT in blood to balance destructive effects of proteases, specifically neutrophil elastase [4, 10]. It has been proposed that therapy with plasma purified A1AT not only normalizes levels of A1AT to inhibit neutrophil elastase activity but also reduces transcriptional levels of neutrophil elastase, and thus may indirectly reduce expression of defective Z-A1AT [11]. Indeed, exogenous A1AT therapy is not recommended for A1ATD patients with liver diseases and its effect on A1ATD liver has not been tested. Therefore, in this study we investigate the potential effects of purified A1AT on SERPINA1 expression in human hepatocytes, circulating mononuclear cells and lung tissue.

## Materials and methods

### SNP analysis and genotyping

The included individuals were genotyped using Single Nucleotide Polymorphism (SNP) genotyping TaqMan^®^ assays on a ABI Step-One Plus (Applied Biosystems) real-time PCR instrument using the allelic discrimination method according to manufacturer’s instruction [12]. All probes were pre-designed SNP Genotyping Assay and purchased from Applied Biosystems. For PiS, the rs1758 (assay ID: C_594695_20) probe was used; for PiZ allele, the rs28929474 (assay ID: C_34508510_10) was used; for the PiM2/M4 allele, the rs709932 (assay ID: C_2895146_20) probe was used and finally for the PiNull allele, rs28929473 (assay ID: C_63321235_20) was used.

### Hepatocyte isolation and culture

With approval from the local ethics committees (Dnr: 2010/678-31/3) liver tissue used for hepatocyte isolation was acquired with informed written consent from patients undergoing liver resection surgery following primary/secondary tumors or explanted livers from A1ATD patients being liver transplanted. Also one of the control liver tissues was obtained from one donor liver unsuitable for transplantation. The isolation process followed previously established and standardized method of three-step collagenase perfusion technique [13, 14]. Cell recovery and viability were determined using trypan blue exclusion method. Isolated primary human hepatocytes were cultured on matrigel matrix derived from Engelbreth-Holm-Swarm sarcoma [15]. Cells were cultured in William’s E medium (Sigma-Aldrich, Stockholm, Sweden) supplemented with HEPES (25mM), glutamine (2mM) insulin (10mM), dexamethasone (10mM), amphotericin B and gentamicin and plated in 12 well plates (Corning Life Sciences, Tewksbury, MA) at a density of 7.5x10^5^ cells per well. The hepatocytes were cultured under standard culture conditions in 37°C incubator with 5% CO_2_.

### A1AT treatment of primary human hepatocytes

Hepatocytes were isolated and plated day 0. The culture plates were divided into two groups, the first group was only treated with purified A1AT and the second group was treated with purified A1AT and subsequently co-treated with 10 ng/ml OSM (PeproTech inc. Rocky Hill, NJ, USA) the final 24 hours of culture. Treatment with purified A1AT (Prolastina^®^; Grifols, Barcelona, Spain) was started day 1 of culture and continued through day 5. The purified A1AT (final well concentration of 1, 3.5, 7 and 10 mg/ml) was mixed together with culture media and used to treat the cells daily.

### Human lung tissue

Surgical lung explants from end-stage PiZZ A1AT emphysema cases with A1AT augmentation therapy (n = 10) and without A1AT therapy (n = 4) were retrieved from the Institute of Pathology of Hannover Medical School with the approval of the Hannover Medical School Ethics Committee (No. 2702–2015).

Gene expression analysis was performed as described previously [16–18]. Briefly, after immunohistochemical and histological evaluation, the specimens were cut into 10μm thick sections and suspended in a proteinase K digestion solution. After overnight incubation RNA was isolated using phenol-chloroform extraction, followed by ethanol precipitation [16]. cDNA was generated from 10μl of RNA using the High Capacity cDNA Reverse Transcription Kit (Applied Biosystems), as described in the manufacturer’s guidelines. Following, cDNA was preamplified with nonrandom PCR primers and PreAmp MasterMix (Applied Biosystems) to lower CT values by 14 PCR cycles [19]. For the subsequent quantitative real-time PCR (TaqMan 7500 Real-Time PCR system, (Applied Biosystems)), the preamplified cDNA was equilibrated with TaqMan Gene Expression Master Mix and the individual TaqMan Gene Expression mixture. Total expression of SERPINA1 was analysed using expression of the gene DNA-directed RNA polymerase II subunit RPB1 (Polr2a/POLR2A) as an endogenous reference gene.

### Human adherent PBMCs

Peripheral blood mononuclear cells (PBMCs) were isolated from peripheral blood of five healthy volunteers using Lymphosep discontinuous gradient centrifugation, according to the manufacturer’s instructions as previously described [20]. PBMCs were resuspended in RPMI 1640 with 2 mM N-acetyl-L-alanyl-L-glutamine (Gibco, Thermo Fisher Scientific, Grand Island, NY) containing supplements of 1% nonessential amino acids (Gibco), 2% sodium pyruvate (Gibco), and 20 mM HEPES (Gibco) and plated at a density of 6×10^6^ cells/ml (12 well plates). Cells were incubated for 75 min at 37°C and 5% CO_2_ to allow monocytes to adhere to the cell culture plates. Afterwards, non-adherent cells were removed by washing with 1X DPBS containing Mg2+ and Ca2+ (Life Technologies), and fresh medium without FCS was added for 24 hours. Adherent PBMCs were treated with 1mg/ml A1AT (Zemaira, CSL Behring, King of Prussia, PA) alone or together with LPS (1μg/ml) (Sigma) for additional 24 hours.

### mRNA expression

Total RNA was extracted using the TRIzol^®^ reagent (Invitrogen, Carlsbad, CA) or the RNeasy Mini Kit (Qiagen, Hilden, Germany), according to the manufacturer’s instructions. RNA was reverse-transcribed using the high-capacity cDNA reverse transcription kit (Applied Biosystems, Thermo Fisher Scientific, Carlsbad, CA) into cDNA. The cDNA was diluted and mixed with TaqMan^®^ assays specific for cyclophilin A (PPIA, Hs99999904_m1), HPRT (Hs02800695_m1) A1AT (SERPINA1, Hs01097800_m1 or Hs00165475_m1) CYP7A1 (CYP7A1, Hs00167982_m1), AFP (AFP, Hs00173490_m1), Ki67 (MKI67, Hs01032443_m1), pcna (PCNA, Hs00427214_g1) and caspase-3 (CASP3, Hs00234387_m1) containing primers and hydrolysis probes. Quantitative real-time PCR was performed on an ABI Step-One Plus (Applied Biosystems) instrument. The relative mRNA expression was calculated according to the ΔΔ cycle threshold method using StepOne software with HPRT as reference gene or by a modified ΔΔ cycle threshold method using cyclophilin A (PPIA) as a reference gene as described in [21].

### Human liver tissue

Paraffin embedded tissue from 6 explanted PiZZ livers was collected and mounted in a tissue array system. Also, liver tissue from 13 previous cases of explanted or resected material was used as an A1AT proficient reference. All sections were stained for Ki67 by automated systems at the Karolinska University Hospital, the Department of Pathology. Antibodies were visualized using diaminobenzidine and sections were counterstained with hematoxylin. At least three or as many as necessary fields to surpass 1000 cells were counted per case. For all cases Ki67 positive hepatocytes and non-parenchymal cells (NPC) were identified and counted. Average number of hepatocytes counted per case was 1405 cell for proficient cases (n = 13) and 1273 for deficient cases (n = 6). Percentage Ki67 positive hepatocytes was calculated as Ki67 positive hepatocytes in all fields divided by all counted hepatocytes per case.

### Statistical analysis

Statistical analysis was performed in Prism version 6 (Graphpad Software Inc., San Diego, CA, USA). Non-parametric Kruskal-Wallis one-way ANOVA on ranks was used for multiple group comparisons. Mann-Whitney *U* test for comparison between two groups or Wilcoxon test where applicable as normality could not be assumed. Values are reported as median with range. P values below 0.05 were considered significant and n values are reported in figures. Bars in boxplots represent medians and + symbol represent mean.

## Results

In total, liver tissue from 15 individuals (7 females and 8 males) was used for hepatocyte isolation Table 1. The median age was 60.5 years (range 18–64, n = 6) for the deficient group and 44 years (range 5–76, n = 9) for the proficient group. Four patients underwent liver transplantation due to A1ATD. One patient with a homozygous ZZ mutation and one patient previously diagnosed as homozygous ZZ using isoelectric focusing were identified. Moreover, two samples were heterozygous Z. Additionally, one patient had a heterozygous S mutation and underwent liver transplantation. Remaining A1AT proficient samples showed no amplification for the Z or S mutations.

**Table 1:** Characteristics of liver tissues used for cell isolation.

Sample	Age	Sex	Diagnosis	Genotype
1	60	F	A1ATD (Explant)	ZZ
2	64	F	A1ATD (Explant)	ZZ
3	18	F	A1ATD (Explant)	ZZ
4	62	M	A1ATD (Explant)	Z_
5	22	M	Organ donor	Z_
6	61	M	A1ATD (Explant)	S_
7	50	F	CRC metastasis	Not Z
8	68	M	CRC metastasis	Not Z
9	36	F	CRC metastasis	Not Z
10	76	F	Renal cancer	Not Z
11	59	M	Alcohol cirrhosis	Not Z
12	35	M	HCC	Not Z
13	5	F	PFIC2	Not Z
14	44	M	Organ donor	Not Z nor S
15	15	M	Crigler-Najjar (Explant)	Not Z nor S

Abbreviations: F, female; M, male; PFIC2, progressive familial intrahepatic cholestasis, type 2; CRC, Colorectal cancer; HCC, Hepatocellular carcinoma

### Purified A1AT decreases the mRNA expression of SERPINA1 in primary human hepatocytes

**Fig 1:**
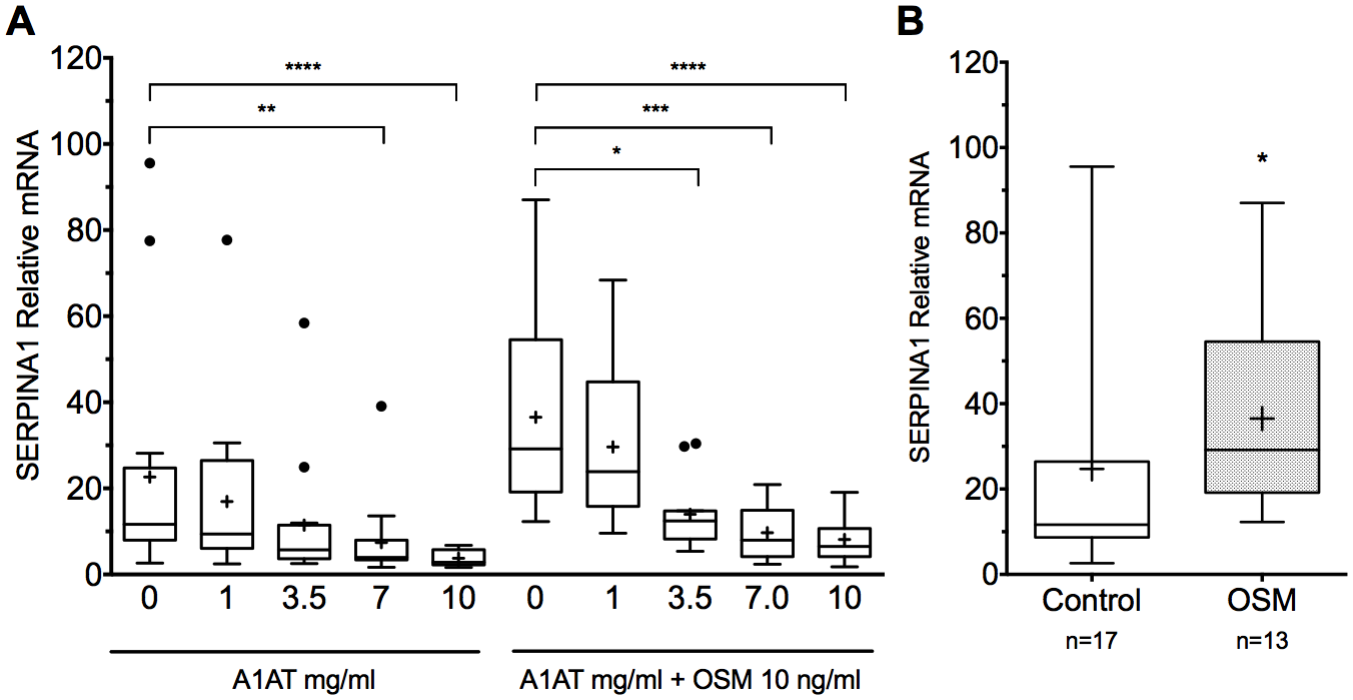
Purified A1AT reduces SERPINA1 expression in a dose-dependent manner in primary human hepatocytes. (**A**) Exogenously added purified A1AT reduced SERPINA1 expression in primary human hepatocytes isolated from both proficient and deficient liver tissue. The reduction was more prominent following Oncostatin M (10ng/ml) stimulation (Kruskal-Wallis test on ranks). SERPINA1 gene expression was reduced in a dose-dependent manner with marked decrease at A1AT levels of >1 mg/ml (**B**) Oncostatin M showed increased expression of SERPINA1 in primary human hepatocytes (Mann-Whitney *U* test), * P<0.05, ** P <.001, *** P<0.001, **** P<0.0001).

To evaluate the putative effect of exogenous A1AT, mRNA from proficient and deficient hepatocytes treated with A1AT was analyzed for SERPINA1 expression levels and compared to untreated controls. As shown in (Fig 1A) a significant decrease was detected in SERPINA1 mRNA expression following treatment with exogenous purified A1AT compared to untreated cells. Exogenous A1AT reduced SERPINA1 expression in a dose-dependent manner.

To further analyze the effects of purified A1AT on hepatocytes with high expression of SERPINA1—OSM a potent inducer of SERPINA1 was employed. Addition of OSM resulted in an increase of SERPINA1 expression (P = 0.0154, Mann-Whitney *U* test) (Fig 1B). The highly up-regulated SERPINA1 was reduced using purified A1AT. Notably, the effect of exogenous A1AT on SERPINA1 expression was more pronounced in OSM-activated hepatocytes than in unstimulated cells.

### SERPINA1 mRNA in lung tissue from PiZZ patients with and without augmentation therapy

**Fig 2:**
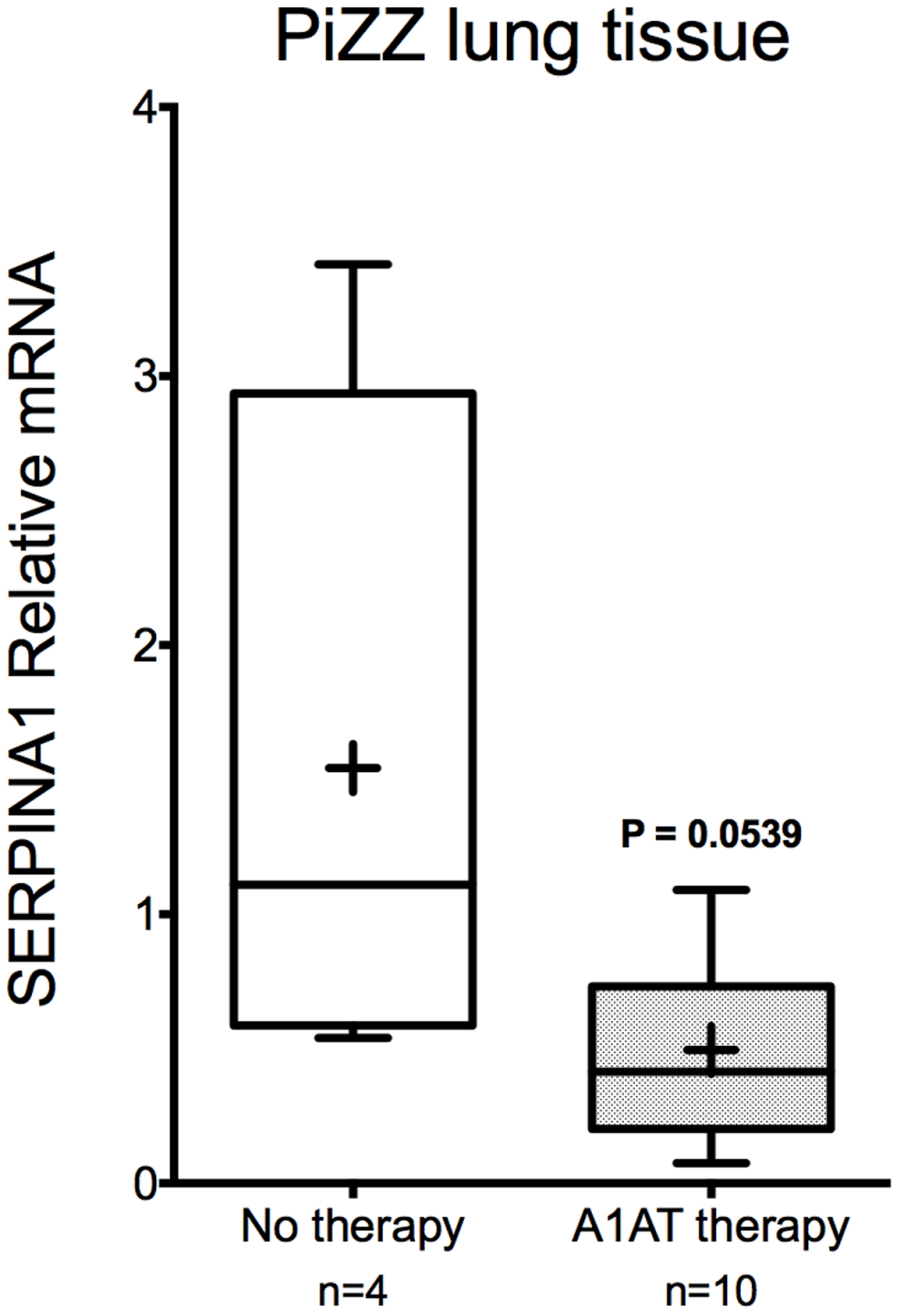
Effect of purified A1AT therapy on SERPINA1 gene expression in the PiZZ lung. SERPINA1 gene expression in lung tissue from PiZZ patients receiving purified A1AT (Prolastin) therapy (n = 10) compared to PiZZ patients without therapy (n = 4) showed similar tendencies to other cell types investigated. There might be reduced SERPINA1 expression in treated PiZZ patients, the difference did however not reach statistical significance (P = 0.0539, Mann-Whitney *U* test).

We questioned if a similar reduction in SERPINA1 gene expression can be observed in lung tissue from A1AT treated PiZZ emphysema patients as we found in hepatocyte cultures. As shown in (Fig 2) median expression of SERPINA1 was 0.42 (range 0.075–1.09) in lung tissue samples from PiZZ patients with augmentation therapy (n = 10) compared to patients without therapy 1.12 (range 0.54–3.42) (n = 4), although, this difference did not reach statistical significance (Median, P = 0.0539, Mann-Whitney *U* test).

### Effects of exogenous A1AT protein on SERPINA1 mRNA in adherent PBMCs, *in vitro*

**Fig 3:**
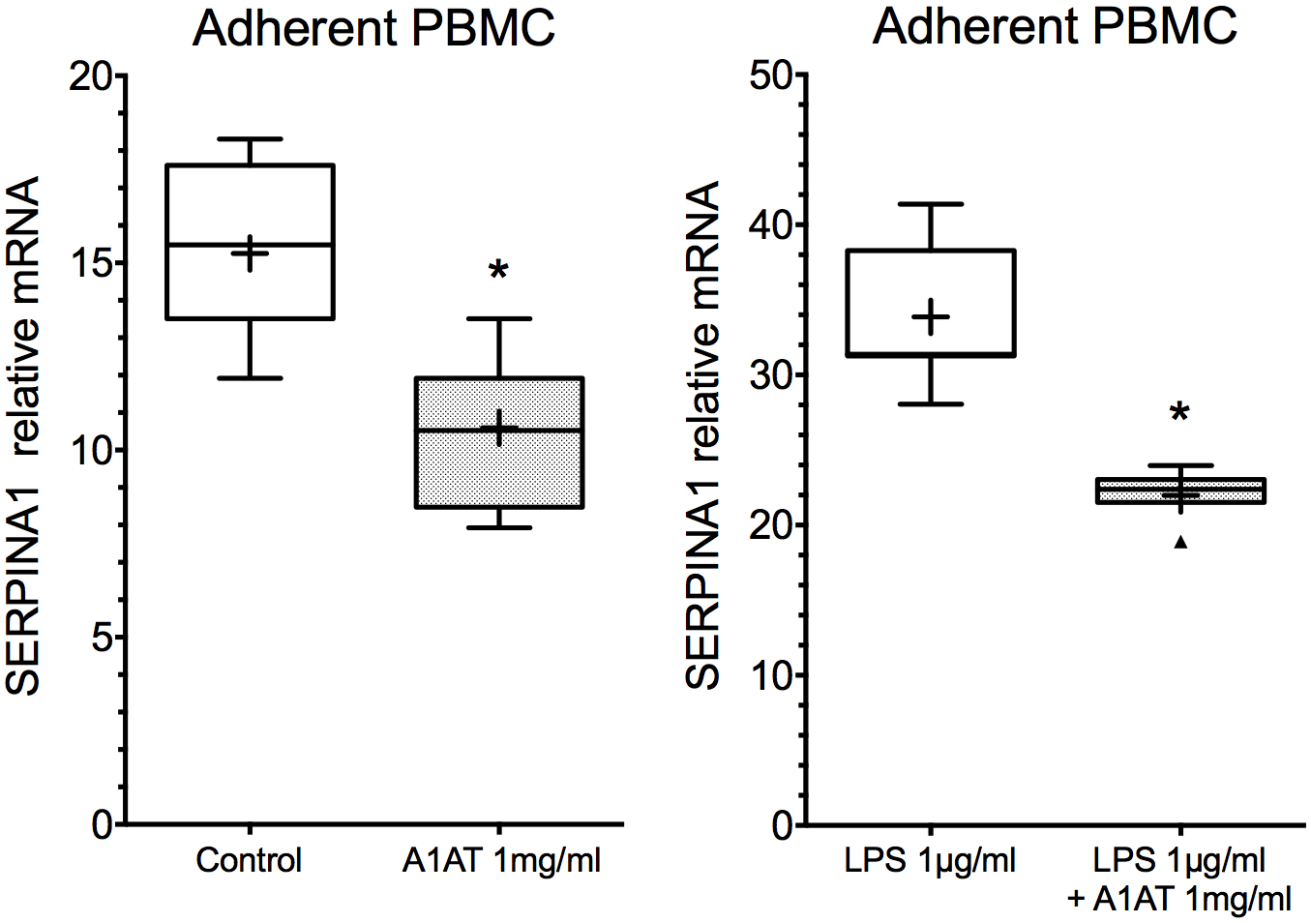
Purified A1AT reduces SERPINA1 expression in adherent PBMCs *in vitro*. Adherent PBMCs isolated from healthy donors showed a reduction in SERPINA1 expression following purified A1AT protein treatment. The reduction could be observed in both naïve and LPS-stimulated cells and was in coherence with the effects seen in hepatocytes (n = 7, Wilcoxon test), * P<0.05.

Investigation of a different cell type other than hepatocytes was carried out to test whether the inhibitory effect of exogenous A1AT on SERPINA1 is a more general phenomenon (Fig 3). Similarly as in hepatocytes, treatment of PBMCs with purified A1AT (1mg/ml) reduced SERPINA1 mRNA expression from 15.48 (range 11.92–18.31) to 10.52 (range 7.92–13.51) (median, n = 7, P = 0.0156, Wilcoxon test). Furthermore, purified A1AT reduced SERPINA1 expression from 31.38 (28.05–41.37) to 22.39 (18.91–23.96) in LPS-stimulated PBMCs. Indeed, effect of exogenous A1AT on SERPINA1 expression was again more pronounced in activated PBMCS than in unstimulated cells (median, (range), n = 7, P = 0.0156, Wilcoxon test).

### mRNA expression in A1AT-deficient vs. -proficient hepatocytes

**Fig 4:**
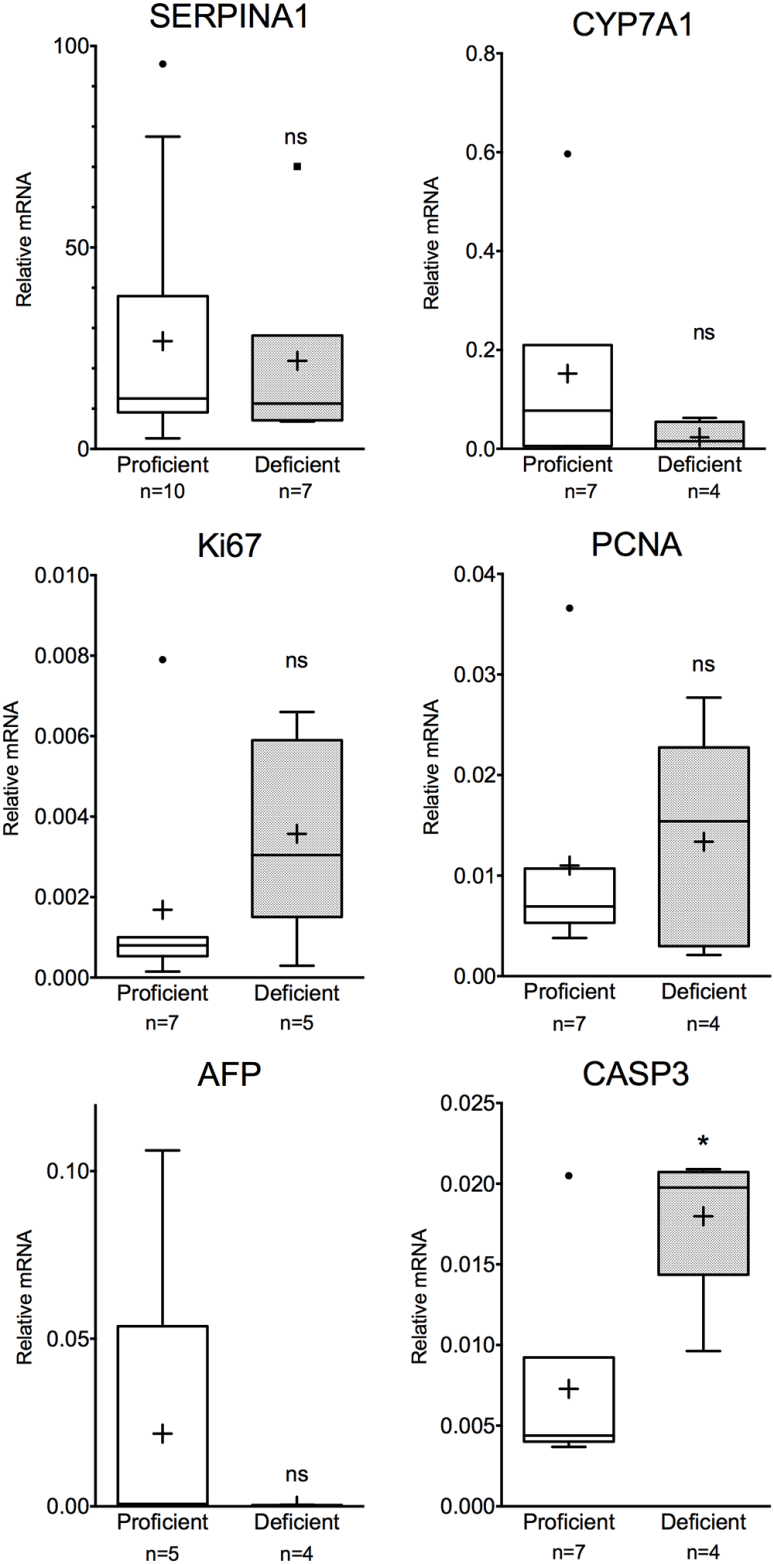
Proficient versus deficient primary human hepatocytes. RT-PCR **e**xpression of several genes showed no remarkable difference between proficient and deficient primary human hepatocytes. SERPINA1 expression was at equal levels in both deficient and proficient hepatocytes, Ki67 levels differed between the groups, although this was not significant. However, expression of CASP3 was increased in A1AT deficient hepatocytes (Mann-Whitney *U* test) * P<0.05, ns not significant.

It has been speculated that there is a higher hepatocellular turnover in a mouse model of A1AT deficiency [22]. Therefore, comparison between A1AT-deficient and proficient hepatocytes was conducted regarding basal levels of SERPINA1 and upregulation of cell turnover. As shown in (Fig 4), no differences were detected in mRNA expression of SERPINA1, CYP7A1, KI67, PCNA and AFP between proficient and deficient cells. However, the expression of CASP3 was higher in deficient cells (P = 0.0177, Mann-Whitney *U* test).

### Liver immunohistochemistry

**Fig 5:**
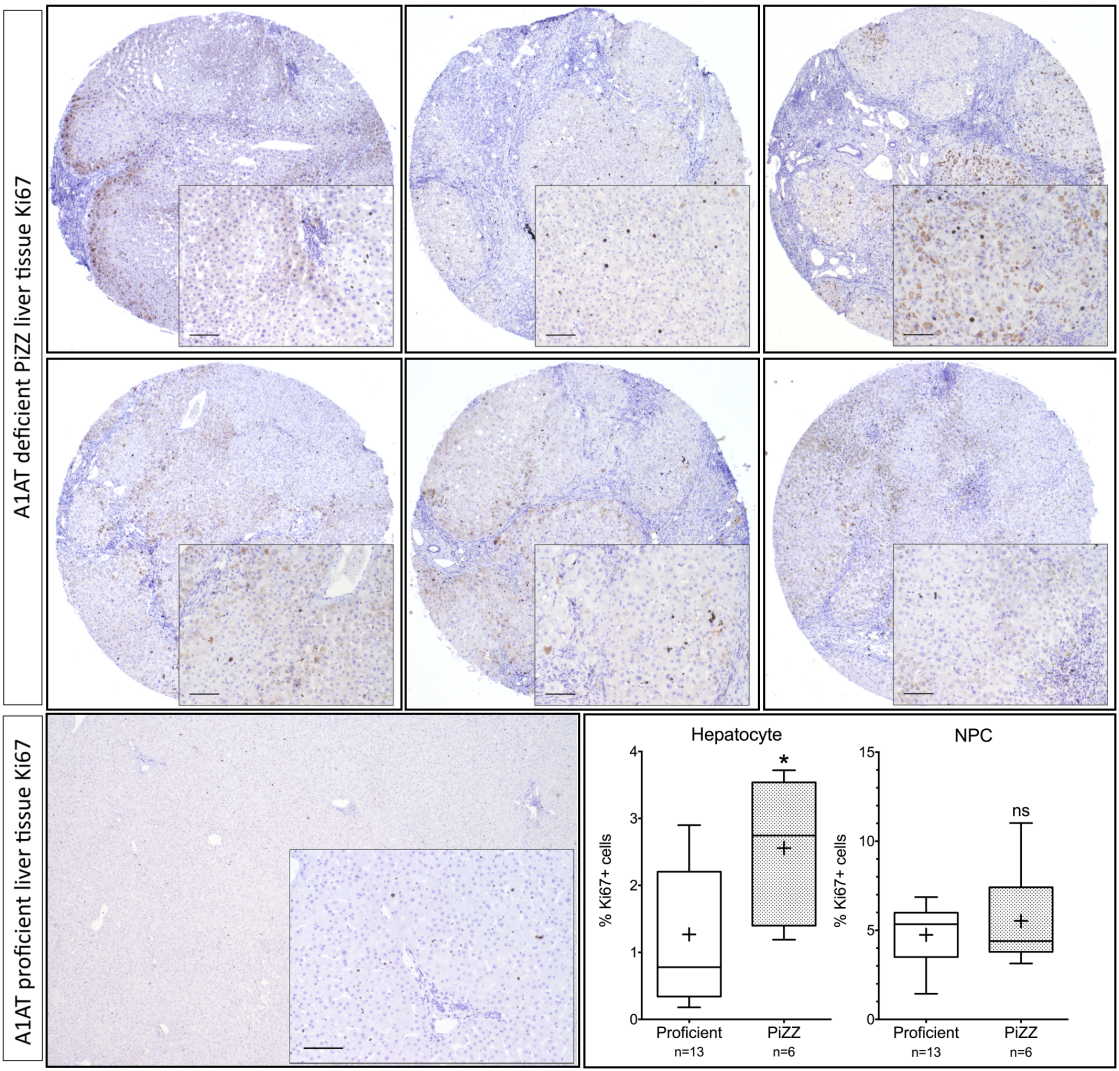
Increased rate of proliferation in the PiZZ liver. Liver tissue from PiZZ patients (n = 6) was stained for proliferation using Ki67 antibody and visualized by diaminobenzidine. Liver tissue from A1AT proficient individuals (n = 13) was used as reference. PiZZ livers show increased frequency of Ki67 positive (proliferating) cells compared to the proficient group. No differences could be observed in the non-parenchymal cells (NPC). (Mann-Whitney *U* test) * P<0.05, ns not significant. Scale bars: 100μm.

Liver tissues from six explanted PiZZ livers were collected and cell proliferation was measured by Ki67 staining in comparison to A1AT proficient tissues. In the PiZZ group the percentage of hepatocytes positive for Ki67 was 2.75% compared to 0.78% in the proficient group (median, P = 0.0285, Mann-Whitney *U* test) (Fig 5). Non-parenchymal cells showed no difference.

## Discussion

Our data provide new evidence that exogenous purified human plasma A1AT protein can lower expression of SERPINA1 gene in human primary hepatocytes and human adherent PBMCs, *in vitro*. Noticeably, this effect of exogenous A1AT was observed in hepatocytes isolated from both A1AT proficient and deficient patients as well as in human PBMCs from PiMM healthy individuals (Fig 3). Similar trends were also detected in lung tissues obtained from a small cohort of PiZZ A1ATD emphysema patients receiving augmentation therapy relative to those without therapy (Fig 2). It is also important to point out that inhibitory effect of exogenous A1AT on SERPINA1 expression was even more pronounced in cells activated with inflammatory stimuli.

Altogether, our findings highlight the more complex benefits of A1AT therapy than just ameliorating protease imbalance in A1AT deficiency. Moreover, our data support the hypothesis that therapy with A1AT not only augments missing protein levels in PiZZ patients but also reduces expression of defective Z-A1AT protein. This may have important implications during acute phase reactions when increased synthesis of Z-A1AT provides favorable conditions for defective Z-A1AT polymerization and intracellular accumulation.

Expression of selected genes reflecting function, damage and proliferation between proficient and deficient cells were similar. There was a large overlap of SERPINA1 expression between proficient and deficient cells suggesting that production of mutant Z-A1AT is equal to non-mutant M-A1AT protein. This latter confirms that Z-A1AT pathologies involving reduced protein secretion due to retention of mutant protein rather than its impaired production [23]. On the other hand, we found a higher CASP3 expression in A1AT deficient cells compared to proficient cells in line with results reported from PiZ transgenic mice [22]. Higher CASP3 expression can be indicative of enhanced apoptotic activity and turnover among the deficient cells resulting from Z-A1AT protein aggregation. In murine models, it has been reported that A1AT could ameliorate acute liver failure through decreased apoptosis and reduced caspase-3 activity [24]. A1AT has also displayed pro-survival and anti-apoptotic effect through caspase-3 inhibition in murine lung endothelial cell, reducing alveolar wall destruction [25]. These are important factors to consider in the clinical setting where restoration of adequate levels of circulating A1AT could alleviate cellular damage.

Hepatocyte proliferation was examined in explanted liver tissue from PiZZ patients undergoing liver transplantation. The extent of cirrhosis was evident. The pathology and cirrhosis was coupled with increased proliferation as revealed by Ki67 staining. These results are consistent with findings from male PiZ transgenic mice, where increased hepatocellular proliferation has been reported [22]. Elevated levels of CASP3 and increased proliferation evident by Ki67 staining in the PiZZ deficient liver are pointing towards a higher cell turnover. We speculate that the deficient PiZZ liver is a suitable candidate for hepatocyte transplantation as the increased cell turnover can be beneficial for repopulation by transplanted healthy PiMM hepatocytes. Transplanting a small fraction of PiMM donor hepatocytes in A1ATD individuals could potentially downregulate the production of Z-A1AT. Functioning circulating M-A1AT has been reported in a hepatocyte transplanted patient with A1AT deficiency [26]. Donor cells would experience a growth advantage as compared to native cells producing faulty protein [22].

The efficacy of augmentation therapy has been debated and focus has mainly been reserved for its protective effect on the lungs. The effects of augmentation therapy on liver status are generally not explored. Although A1ATD patients report a subjective improvement and better quality of life, hesitation in initiating the therapy and difficulties justifying the high cost and lifelong repeated hospital visits for intravenous treatment remain [1, 27]. In this context, studies of hepatic effects of augmentation therapy are important in understanding unknown aspects of the disease and therapies.

Small groups were a hindrance in statistical evaluations. Another limitation is the absence of Z-A1AT levels in culture media following treatment with M-A1AT (Prolastina^®^). We were not able to separate between exogenously added M-A1AT and Z-A1AT released by hepatocytes in culture media. Effort can be directed toward studies of SERPINA1 expression and A1AT content in liver biopsies from patients receiving augmentation therapy. Unfortunately, such material was not available to us.

Although our results are preclinical, if confirmed by further studies, they could have several implications for A1ATD patients. New long term treatment regiment aiming at reducing or preventing liver disease, as well as lung disease could have a tremendous impact on health care for A1ATD patients.

## Conclusions

In conclusion, our data provide evidence that plasma purified A1AT can reduce SERPINA1 gene expression. This effect was observed in both deficient and proficient cells suggesting a beneficial effect of augmentation therapy for liver that should be evaluated further.
